# African walnut (*Plukenetia conophora*) oil improves glucose uptake and metabolic activities in erythrocytes

**DOI:** 10.3389/fnut.2025.1607386

**Published:** 2025-07-09

**Authors:** Ochuko L. Erukainure, Chika I. Chukwuma

**Affiliations:** ^1^Laser Research Centre, Faculty of Health Sciences, University of Johannesburg, Johannesburg, South Africa; ^2^Centre for Quality of Health and Living (CQHL), Faculty of Health and Environmental Sciences, Central University of Technology, Bloemfontein, South Africa

**Keywords:** African walnut, erythrocytes, glucose metabolism, oil, oxidative stress

## Abstract

**Background:**

African walnut (*Plukenetia conophora*) oil (AWO) has been employed in the management of glucose dysmetabolic-mediated ailments, with emerging evidence suggesting that its modulatory effects on erythrocyte glucose dysmetabolism may mitigate dysfunctions implicated in the pathophysiology of metabolic diseases.

**Objective:**

The present study investigated the effect of AWO on glucose uptake and its effect on glucose metabolism, purinergic and antioxidant activities and surface morphology in isolated rats’ erythrocytes *ex vivo*.

**Methods:**

Isolated erythrocytes were incubated with AWO (30–240 μg/mL) and glucose (11.1 mM) for 2 h at 37°C. Negative control consisted of erythrocytes incubated with glucose only, while normal control consisted of erythrocytes not incubated with AWO and/or glucose. Metformin served as the standard hypoglycemic drug.

**Results and conclusion:**

Incubation with AWO led to significant increase in erythrocyte glucose uptake, with concomitant suppression in superoxide dismutase, adenosine triphosphatase, ecto-nucleoside triphosphate diphosphohydrolase, glucose 6-phosphatse and fructose-1,6-bisphosphatase activities and iron level, while concomitantly enhancing glutathione and magnesium levels. Furthermore, the surface morphology of erythrocytes was improved following incubation with AWO. Molecular docking analysis revealed strong molecular interactions between AWO’s phytoconstituents (linolenic acid and linoleic acid) and hemoglobin. Molecular dynamics simulation further revealed strong protein-ligand relationships between hemoglobin the oil’s constituents as revealed by root mean square deviation, root mean square fluctuation, solvent accessible surface area, and radius of gyration values, with hydrogen, hydrophobic, ionic bonds and water bridges contributing to the stability of the protein-ligand complex. These results suggest the ability of AWO to improve erythrocyte glucose metabolism and morphology, mitigate oxidative stress, and may be of translational relevance in managing erythrocytes’ dysfunction in metabolic diseases.

## 1 Introduction

Erythrocytes are also known as red blood cells, and are blood components involved in the binding, transportation and release of oxygen (O_2_) and carbon dioxide (CO_2_). These roles have been attributed to their flexibility which allows them to move freely through capillaries and the presence of the main oxygen-carrying protein, hemoglobin (1, 2). The morphology of erythrocytes also plays a major role in their survival and function, as alteration its normal physiological biconcave discoid shape has been implicated in the pathophysiology of several diseases including diabetes, and sickle cell disease (1, 3, 4). Erythrocytes depend on glucose as their primary source of energy, which is anaerobically metabolized via the glycolytic pathway to generate ATP (5). Erythrocyte glucose uptake and metabolism are important for their function and survival, and disturbances have been implicated in alterations of their morphology, O_2_ transportation and half-life (3, 6). These alterations affect the formation of clots, capillary functions and blood flow leading to an elevated risk of thrombotic episodes or vascular problems (7, 8). Oxidative stress arising from increased generation of reactive oxygen species (ROS) and free radicals, and impaired antioxidant defense system, is among the pathophysiology of altered erythrocyte glucose metabolism (6, 9). Targeting erythrocyte glucose uptake and metabolism may present a therapeutic strategy in managing erythrocyte dysfunctions in diseases such as diabetes where it has been implicated in its complications (3, 8).

African walnuts (*Plukenetia conophora*) are underutilized nuts indigenous to tropical western and central Africa, and belong to the Euphorbiaceae family (10). They have been reported for their nutritional and health benefits with emphasis on their high oil content (11). African walnut oil (AWO) has been reported for its antioxidant properties which is demonstrated by its ability to improve superoxide dismutase (SOD) and catalase activities, while maintaining hepatic morphology in sodium arsenate induced oxidative hepatic injury (12). The oil decreased serum levels of LDL-cholesterol, triglyceride and cholesterol, while modulating the hepatic biomarkers, alkaline phosphatase (ALP), aspartate aminotransferase (AST) and alanine aminotransferase (ALT) in normal male albino rats and individuals with type 2 diabetes (13, 14). The oil also suppressed fasting blood glucose level in individuals with type 2 diabetes (14). Recently, we demonstrated the ability of AWO to promote glucose uptake and improve carbohydrate and energy metabolism as well as other biological activities linked to male fertility in testicular tissues (15). These biological activities of AWO have been attributed to its phytochemical constituents which include linoleic acid (39.0%), linolenic acid (42.89%), 9-hexadecenoic acid (01.1%), oleic acid (0.27%), oleic anhydride (3.75%), eicosanoic acid (4.1%), cis-5-dodecenoic acid (0.14%), octadecanoic acid (11.63%) and 2-myristynoic acid (0.13%) (11, 15).

Although the ability of AWO to stimulate glucose uptake, and modulate carbohydrate metabolism and antioxidant activity have been demonstrated in testicular tissues (15), there is still a dearth on its effect on erythrocyte glucose uptake and metabolism. Thus, the present study was carried out to determine the effect of AWO on erythrocyte glucose metabolism by investigating its ability to promote glucose uptake, glucogenic, purinergic and antioxidant activities in isolated rats’ erythrocytes *ex vivo*.

## 2 Materials and methods

### 2.1 Plant material

Fresh African walnut fruits were bought from a local fruit seller in Ore, Ondo State, Nigeria. The fruits were rinsed and their seeds, dehulled. The seeds were airdried, blended and then extracted with hexane. The AWO was obtained by concentrating the hexane extract in a fume hood. The recovered oil was stored in amber glass vials at ambient temperature until further analyses.

### 2.2 Fatty acid profile of African walnut oil

**Figure 1:**
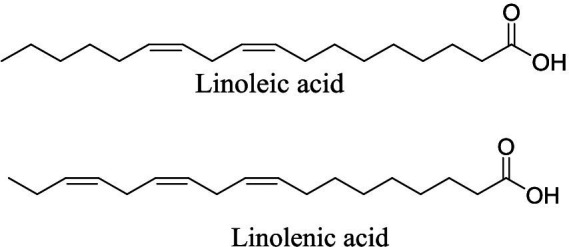
GC-MS identified linoleic acid and linolenic acid in African walnut oil.

The fatty acid constituents of AWO have been previously reported following GC-MS analysis (15). Linoleic acid and linolenic acid (Figure 1) were identified as the predominant fatty acids as they accounted for 39.03 and 42.89% of the total fatty acids, respectively (15).

### 2.3 Animals for *ex vivo* studies

Five male Wister albino rats, weighing between 180 and 250 grams, were procured and kept at the animal house facility located within the Department of Biochemistry at the College of Medicine, University of Lagos, Nigeria. The animals were humanely sacrificed by euthanizing with halothane following an overnight period of fasting. The research was conducted in accordance with the authorized protocol, CMUL/REC/00314.

### 2.4 Extraction of erythrocytes

Blood (8–10 mL) was collected via cardiac puncture into EDTA tubes and centrifuged 10,000 rpm for 10 min at 4°C. The supernatant was discarded and phosphate-buffered saline (PBS) was added to the tubes and centrifuged at 10,000 rpm for 10 min at 4°C to wash the erythrocytes. This was repeated thrice. PBS was added to the erythrocytes and used immediately for glucose uptake study.

### 2.5 Glucose uptake in isolated erythrocytes

A previously described method with slight modifications was used in determining glucose uptake (16). Briefly, 0.5 mL of the freshly harvested erythrocytes was mixed with 7.5 mL with Krebs buffer containing 11.1 mM glucose and different concentrations of AWO (30–240 μg/mL) and incubated for 2 h under the conditions: 95% oxygen and 5% CO_2_, at 37°C. Normal control consisted of reaction mixture incubated without AWO, while metformin served as the standard drug. Glucose concentrations of aliquots (2 mL) collected from the reaction mixtures before and after the incubation were measured with a glucose (GO) Assay Kit (Merck, Johannesburg, South Africa) according to the manufacturer’s manual. Glucose uptake was calculated using the formula:


$$\begin{array}{l} \text{Glucose uptake}\;\mathrm{per}\;\text{volume of}\;\\ \;\;\;\;\;\;\;\;\text{erythrocytes}=\frac{\mathrm{GC}1-\mathrm{GC}2\;}{\text{Volume of erythrocytes}\;(\mathrm{mL})} \end{array}$$

where GC1 and GC2 represent glucose concentrations (mg/dL) before and after incubation, respectively. The glucose concentration in mg/dL was converted to mM by dividing with 18. Glucose uptake was recorded as change in glucose concentration (mM) per mL of erythrocytes.

After glucose uptake assay, the reaction mixture was centrifuged at 10,000 rpm for 10 min at 4°C. The supernatant was discarded and the erythrocytes was resuspended in equal volumes in Eppendorf tubes. About 100 μL of the erythrocytes were freeze-dried and used for electron microscopy analysis (17). About 300 μL of the erythrocytes was mixed with 3,000 mL of PBS (containing 0.5% Triton X-100) and subjected lysis. The lysed cells were centrifuged at 10,000 rpm for 10 min at 4°C. The supernatants were collected into 2 mL Eppendorf tubes and stored at −20°C for further biochemical analyses.

### 2.6 Glucogenic enzymes activities

The erythrocytes were assayed for glucogenic enzymes activities which covers fructose-1,6-bisphosphatase and glucose 6-phosphatase activities using previously described methods (18, 19).

#### 2.6.1 Fructose-1,6-bisphosphatase activity

Briefly, 100 μL of the supernatant was incubated with 100 μL of 0.05 M fructose, 1,200 μL of 0.1 M Tris–HCl buffer (pH 7.0), 250 μL 0.1 M MgCl_2_, 100 μL 0.1 M KCl, and 250 μL 1 mM EDTA at 37°C for 15 min. The reaction was stopped by adding 10% TCA to the reaction mixture and further centrifuged at 3,000 rpm for 10 min (4°C). One hundred microliters of the supernatant was transferred into a 96-well plate. About 50 μL of freshly prepared 9% ascorbic acid and 1.25% ammonium molybdate were then added to the reaction mixture and allowed to stand for 20 min at ambient temperature. Absorbance was read at 680 nm using a microplate reader (SpectraMax M2 microplate reader, Molecular Devices, San Jose, CA, United States). The enzyme activity was extrapolated from an inorganic phosphate (Pi) standard graph generated from sodium phosphate salt (Sigma-Aldrich, Johannesburg, South Africa).

#### 2.6.2 Glucose 6-phosphatase activity

Briefly, 200 μL of the supernatant was incubated with 100 μL of 0.25 M glucose 6-phosphatase, 200 μL of 5 mM KCl, 1,300 μL of 0.1 M Tris–HCl buffer at 37°C in a shaker for 30 min. The reaction was stopped by adding 1 mL of distilled water and 1.25% ammonium molybdate to the reaction mixture. One milliliter of freshly prepared 9% ascorbate was added to the reaction mixture and allowed to stand for 30 min. Absorbance was read at 660 nm using a microplate reader (SpectraMax M2 microplate reader, Molecular Devices, San Jose, CA, United States). The enzyme activity was extrapolated from an inorganic phosphate (Pi) standard graph generated from sodium phosphate salt.

### 2.7 Determination of oxidative stress biomarkers

The erythrocytes were assayed for oxidative stress levels by determining the reduced glutathione (GSH) level and superoxide dismutase (SOD) activities using previously described methods (20, 21).

#### 2.7.1 Reduced glutathione level

Briefly, 200 μL of the supernatant was deproteinized with 10% TCA and centrifuged at 3,500 rpm for 5 min at ambient temperature. One hundred microliters of the resulting supernatant was mixed with 25 μL of Ellman’s reagent in 96 well plate and allowed to stand for 5 min. Absorbance was read at 415 nm with a microplate reader (SpectraMax M2 microplate reader, Molecular Devices, San Jose, CA, United States), and GSH level was extrapolated from a GSH standard curve.

#### 2.7.2 Superoxide dismutase enzyme activity

Fifteen microliters of the supernatant was mixed with 170 μL of 0.1 mM diethylenetriaminepentaacetic acid (DETAPAC) in a 96-well plate. Fifteen microliters of 1.6 mM 6-hydroxydopamine (6-HD) was then added to the mixture. Absorbance was read at 492 nm wavelength for 3 min at 1 min interval with a microplate reader as mentioned previously.

### 2.8 Determination of nucleotide metabolism

The erythrocytes were assayed for nucleotide metabolism by determining the adenosine triphosphatase (ATPase) and ecto-nucleoside triphosphate diphosphohydrolase (E-NTPDase) activities according to previously described methods (22, 23).

#### 2.8.1 ATPase activity

Briefly, 200 μL of the supernatant was incubated with 200 μL of 5 mM KCl, 1,300 μL of 0.1 M Tris–HCl buffer, and 40 μL of 50 mM ATP for 30 min at 37°C in a shaker. One milliliter of distilled water and ammonium molybdate were added to the reaction mixture to stop the reaction. 10% TCA was added to the mixture and allowed to stand on ice for 10 min. Absorbance was read at 660 nm using a microplate reader as mentioned previously. The enzyme activity was extrapolated from an inorganic phosphate (Pi) standard graph generated from sodium phosphate salt.

#### 2.8.2 E-NTPDase activity

Briefly, 20 μL of supernatant was incubated with 200 μL of the reaction buffer (1.5 mM CaCl_2_, 5 mM KCl, 0.1 mM EDTA, 10 mM glucose, 225 mM sucrose and 45 mM Tris–HCl) at 37°C for 10 min. Fifteen microliters of 50 mM ATP was added to the reaction mixture and further incubated in a shaker for 20 min at 37°C. The reaction was stopped by with 200 μL of 10% TCA. Two hundred microliters of 1.25% ammonium molybdate and freshly prepared 9% ascorbic acid was then added to the reaction mixture. The reaction mixture was allowed to stand on ice for 10 min and absorbance was measured at 600 nm with a microplate reader as mentioned previously. The enzyme activity was extrapolated from an inorganic phosphate (Pi) standard graph generated from sodium phosphate salt.

### 2.9 Electron microscopic analysis

#### 2.9.1 Surface morphology

The surface morphology of the erythrocytes was determined by scanning electron microscopy (SEM) analyses. Briefly, about 0.1 g of the freeze-dried samples were placed on the adhesive side of a tape on a stub and gold coated. Images were observed and taken at an accelerating voltage of 20–25 kV with a SEM (Zeiss Ultra Plus) (24).

#### 2.9.2 Energy dispersive X-ray microanalysis

The erythrocytes levels of iron (Fe) and magnesium (Mg) were determined via energy dispersive X-ray (EDX) microanalysis using a SEM (Zeiss Ultra Plus) equipped with an Oxford Instruments X-Max 80 mm2 Solid State EDX detector (24).

### 2.10 Computational studies

To understand the molecular interactions and the ligand-protein relationship of AWO and erythrocytes, its main constituents were subjected to molecular docking and molecular dynamics simulation with hemoglobin.

#### 2.10.1 Protein target selection and preparation

The one-dimensional structures of the protein receptors for hemoglobin was retrieved from the Protein Data Bank (PDB)^1^ using the PDB ID 4HHB. Discovery Studio 2021 was utilized in preparing and refining the protein for docking.^2^ The protein was further converted into nascent receptors by removing the co-crystallized ligand and excess water molecules, which was then followed by the addition of hydrogen and charges.

#### 2.10.2 Molecular docking

The GC-MS identified major compounds (linoleic acid and linolenic acid) (Figure 1) were subjected to molecular docking with 4HHB. The compounds were prepared with MarvinSketch 6.2.1, 2014.

Molegro Molecular Viewer (MMV) and Chem-Axon^3^ were utilized in verifying the accurate representation of the hybridization state and proper angles display of the compounds (25). OPLS4 force field was utilized in the docking using the Schrödinger suite (version 2023-2). The ligand-protein complex was then analyzed and virtualized with BIOVIA Discovery Studio Visualizer and UCSF.

#### 2.10.3 Molecular dynamic simulation

The Desmond module of Schrödinger 2023-2 was used to conduct molecular dynamics simulation (MDS) analysis. The purpose of the MDS was to assess the stability and estimate the dynamic behavior of each complex of 4HHB and the two ligands (linoleic acid and linolenic acid). Briefly, top-scoring docked poses of 4HHB and ligands were prepared for MDS by placing them in a single-point charge (SPC) explicit orthorhombic box with a buffer distance of 10 Å. The system was solvated with a transferable intermolecular potential 3P (TIP3P) water model and neutralized by adding 0.15 M NaCl and Na^+^/ Cl^−^ ions. The long-range electrostatic interactions were calculated with the particle-mesh Ewald method. Short-range van der Waals and Coulomb interactions were cut off at a 9.0 Å radius. OPLS-2005 forcefield (2023) parameters was utilized in minimizing the solvated system and this was followed by relaxation (71). The system was stimulated with the Berendsen NVT ensemble by maintaining pressure (*p* = 1.01325 bar) and temperature (*T* = 300 K) using Nosè–Hoover chain thermostat and Martyna–Tobias–Klein barostat methods, respectively (72). Following the simulation process, the NPT ensemble was initiated with a production run lasting 100 ns. The Centre for High Performance (CHPC, Cape Town) was used in performing MDS workflow remotely.

#### 2.10.4 Post-molecular dynamics simulation analysis

As the trajectories progressed step by step, measurements were taken every 50 ps. The resulting trajectories which cover for protein-stability (RMSD), flexibility (RMSF), radius of gyration (RoG), and solvent-accessible surface area (SASA), were analyzed with an AMBER 20 integrated CPPTRAJ module (26).

#### 2.10.5 Binding free energy analysis

Molecular mechanics in conjunction with the generalized Born surface area (MM-GBSA) approach were used in determining the binding free energies of the complexes. The binding free energy (ΔG_bind_) of MM-GBSA (kcal/mol) was calculated by summing the energy components, columbic, hydrogen bond, van der Waals, self-contact, lipophilic, and solvation of ligand and protein (Equation 1).


(1) $$\Delta G_{\text{bind}}=G_{\mathrm{MM}}+G_{\text{Solv}}-G_{\mathrm{SA}}$$

where ΔG_bind_ = binding free energy, ΔG_MM_ = difference between the free energies of ligand-protein complexes and the total energies of protein and ligand in isolated form, ΔG_Solv_ = difference in the G_SA_ solvation energies of the ligand-receptor complex and the sum of the solvation energies of the receptor and the ligand in the unbound state, ΔG_SA_ = difference in the surface area energies for the protein and the ligand.

### 2.11 Statistical analysis

All biological analyses were carried out in triplicates. Data were analyzed by one-way ANOVA and presented as mean ± SD, with significant difference set at *p* < 0.05 using SPSS version 27 (IBM Corp., Armonk, NY, United States).

## 3 Results and discussion

Glucose plays an important role in the function and survival of erythrocytes and disturbances in its metabolism has been implicated in alteration of its morphology, function and shelf-life (5). These alterations have been linked to several complications including stroke, cardiomyopathy, hypertension and atherosclerosis in diabetes and other metabolic diseases (2, 27, 28). African walnut has been employed in the management of metabolic dysfunctions (14). In the present study, AWO was investigated for its effect on glucose uptake and metabolism, and biological activities linked to erythrocytes dysfunction.

### 3.1 Erythrocyte glucose uptake

**Figure 2:**
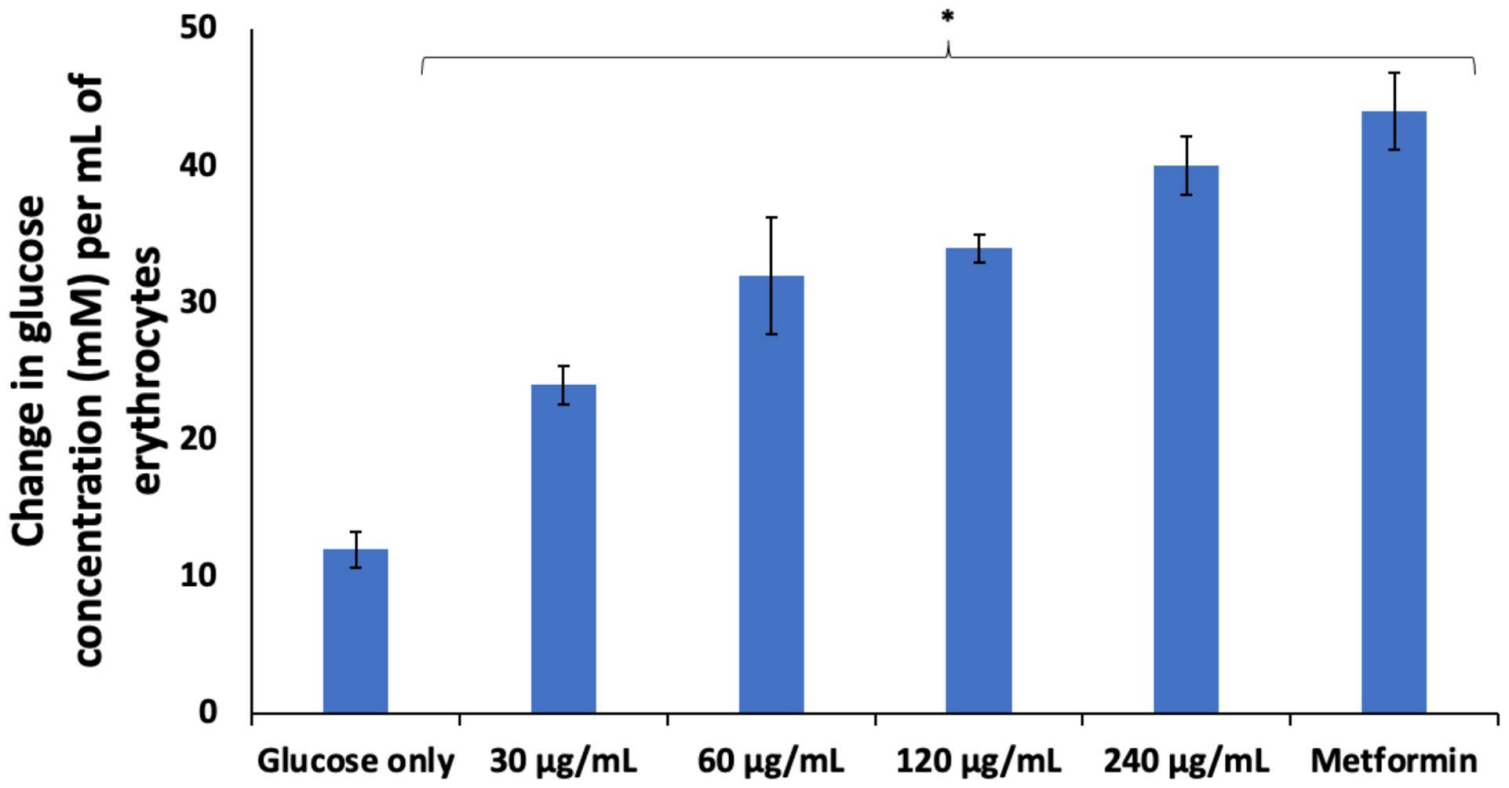
Effect of African walnut oil glucose uptake in erythrocytes. Values = mean ± SD; *n* = 3. ^*^Statistically significant (*p* < 0.05) to glucose only.

The sole dependence of erythrocytes on glucose for energy needed for its function and survival has been well documented (5, 29, 30). Glucose uptake in erythrocytes is essential for its physiology and it is facilitated by glucose transporter 1 (GLUT1) (1, 2). Its impairment has been reported in individuals with diabetes (2, 31, 32). Improving erythrocyte glucose uptake may be a therapeutic target in managing complications linked to erythrocytes dysfunctions in diabetes and other diseases. As shown in Figure 2, AWO significantly (*p* < 0.05) stimulated erythrocytes glucose uptake dose-dependently, and compared favorably with metformin at the highest dose (240 μg/mL). This indicates the ability of AWO to improve erythrocyte glucose uptake and correlates with our previous study on improved glucose uptake by AWO in testicular glucose uptake (15).

### 3.2 Glucogenic enzyme activities

**Figure 3:**
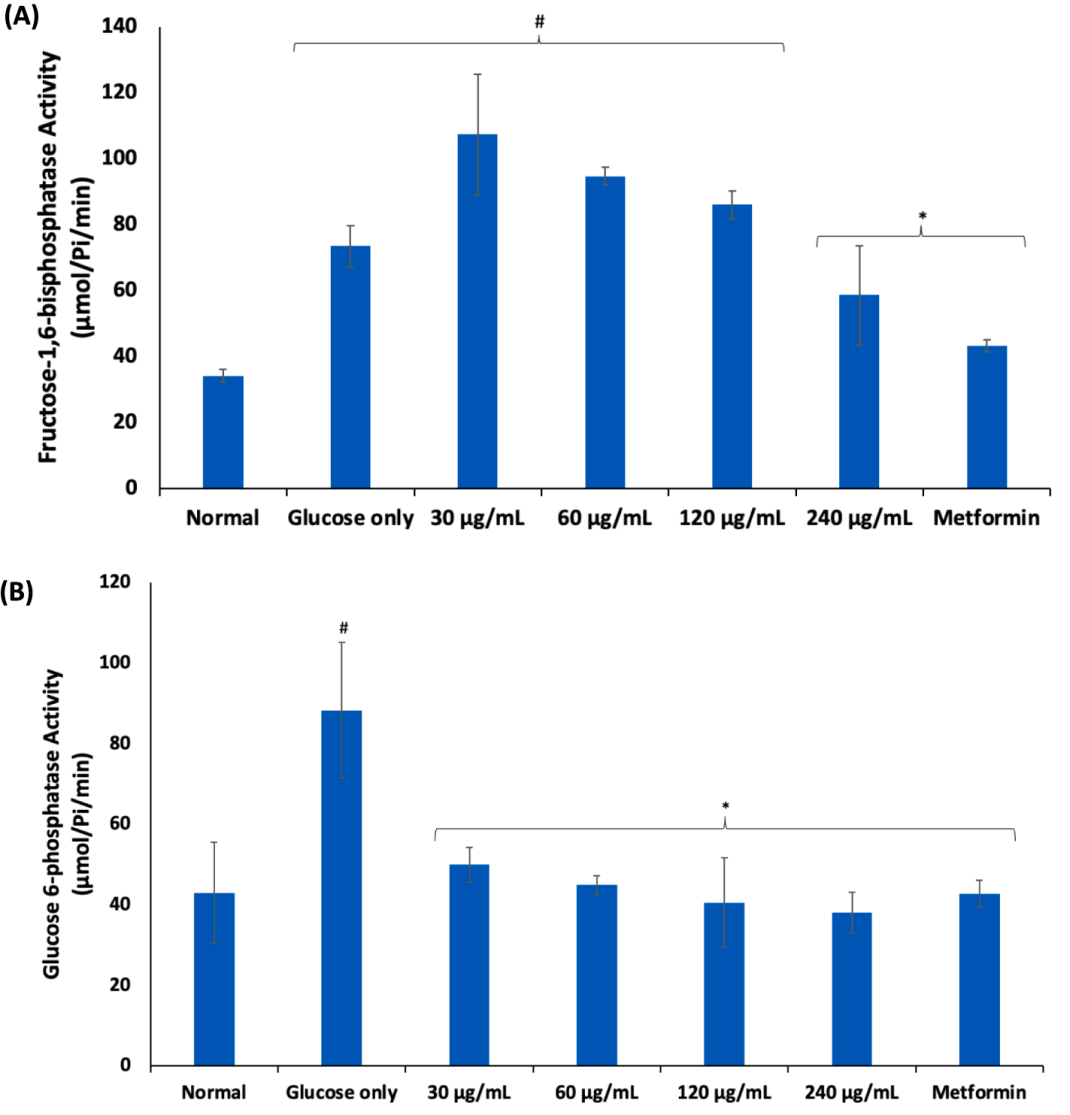
Effect of African walnut oil on **(A)** fructose-1,6-bisphosphatase; and **(B)** glucose 6-phosphatase activities in erythrocyte glucose uptake. Values = mean ± SD; *n* = 3. ^*^Statistically significant (p < 0.05) to glucose only. ^#^Statistically significant (*p* < 0.05) to control.

Following uptake into erythrocytes, glucose is anaerobically metabolized to generate ATP for energy via the glycolytic pathway (5, 6). However, alteration of this pathway characterized by glucose-metabolic enzyme abnormalities has been reported in impaired erythrocyte glucose uptake and transportation as seen in type 2 diabetes (T2D) (5, 30). This is depicted in the present study by the elevated activities of fructose-1,6-bisphosphatase and glucose 6-phosphatase activities in erythrocytes incubated in glucose only (Figure 3). These are key enzymes in the glucogenesis and their elevation may indicate a compensatory switch from glycolysis to glucogenesis to generate glucose for the erythrocyte utilization for ATP generation (33). However, the present study is *ex* vivo and thus, this hypothesis needs to be further investigated as glucogenesis is yet to be reported in erythrocytes. The activities of these enzymes were significantly suppressed following incubation with AWO, indicating restoration of glycolysis in the erythrocytes.

### 3.3 Oxidative stress

**Figure 4:**
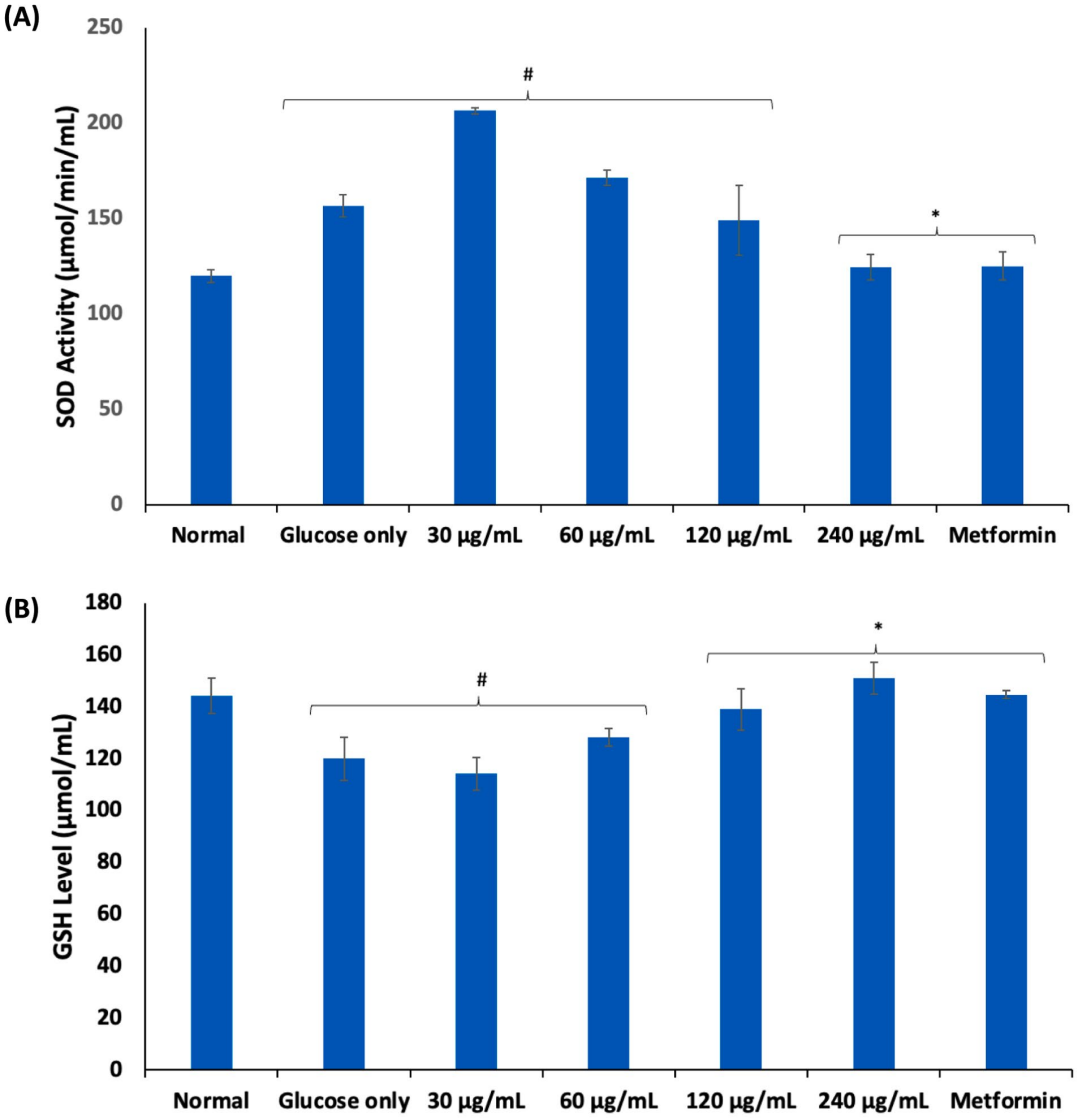
Effect of African walnut oil on **(A)** SOD activity; and **(B)** GSH level in erythrocyte glucose uptake. Values = mean ± SD; *n* = 3. ^*^Statistically significant (*p* < 0.05) to glucose only. ^#^Statistically significant (*p* < 0.05) to control.

Oxidative stress arising from ROS and free radicals have been implicated in erythrocyte dysfunctions (8). Chronic exposure of erythrocytes to high glucose coupled with poor glucose uptake and/or utilization has been implicated in glucotoxicity of the erythrocytes as seen in diabetes (34, 35). Glucotoxicity is characterized by a cascade of biochemical events including generation of ROS, free radicals and suppression of the erythrocytes’ antioxidant defense system (35). In the present study, exposure of erythrocytes to glucose only, led to significant (*p* < 0.05) elevated SOD activity and suppressed GSH level as shown in Figures 4A,B. These alterations depict a compromise in the erythrocytes’ antioxidant defense system and has been reported in diabetics (36–38). The elevated SOD activity indicates high cellular levels of hydrogen peroxide (H_2_O_2_) generated from the enzyme-catalyzed dismutation of superoxide radicals (O_2_^•−^) which might have been generated from the enolization of glucose. The presence of H_2_O_2_ sets up a Fenton reaction where H_2_O_2_ react with hemoglobin to form ferryl hemoglobin (ferrylHb) and oxoferrylhemoglobin (oxoferrylHb) (39). These transient radicals, ferrylHb and oxoferrylHb, have been implicated in the pathogenesis and progression of oxidative stress, leading to cellular damage (40, 41). The low GSH level may be attributed to impaired glucose availability for the GSH generation via the pentose phosphate pathway (PPP) (9). The SOD activity and GSH levels were significantly (*p* < 0.05) reversed in erythrocytes incubated with AWO. These reversions indicate an improvement in the erythrocyte antioxidant defense system and corroborates with previous studies on the ability of the oil to improve antioxidant biomarkers (15). These antioxidant activities may be attributed to the high contents of linolenic and linoleic acids of AWO, which have been reported for their potent antioxidant activities (42, 43).

### 3.4 Purinergic enzyme activities

**Figure 5:**
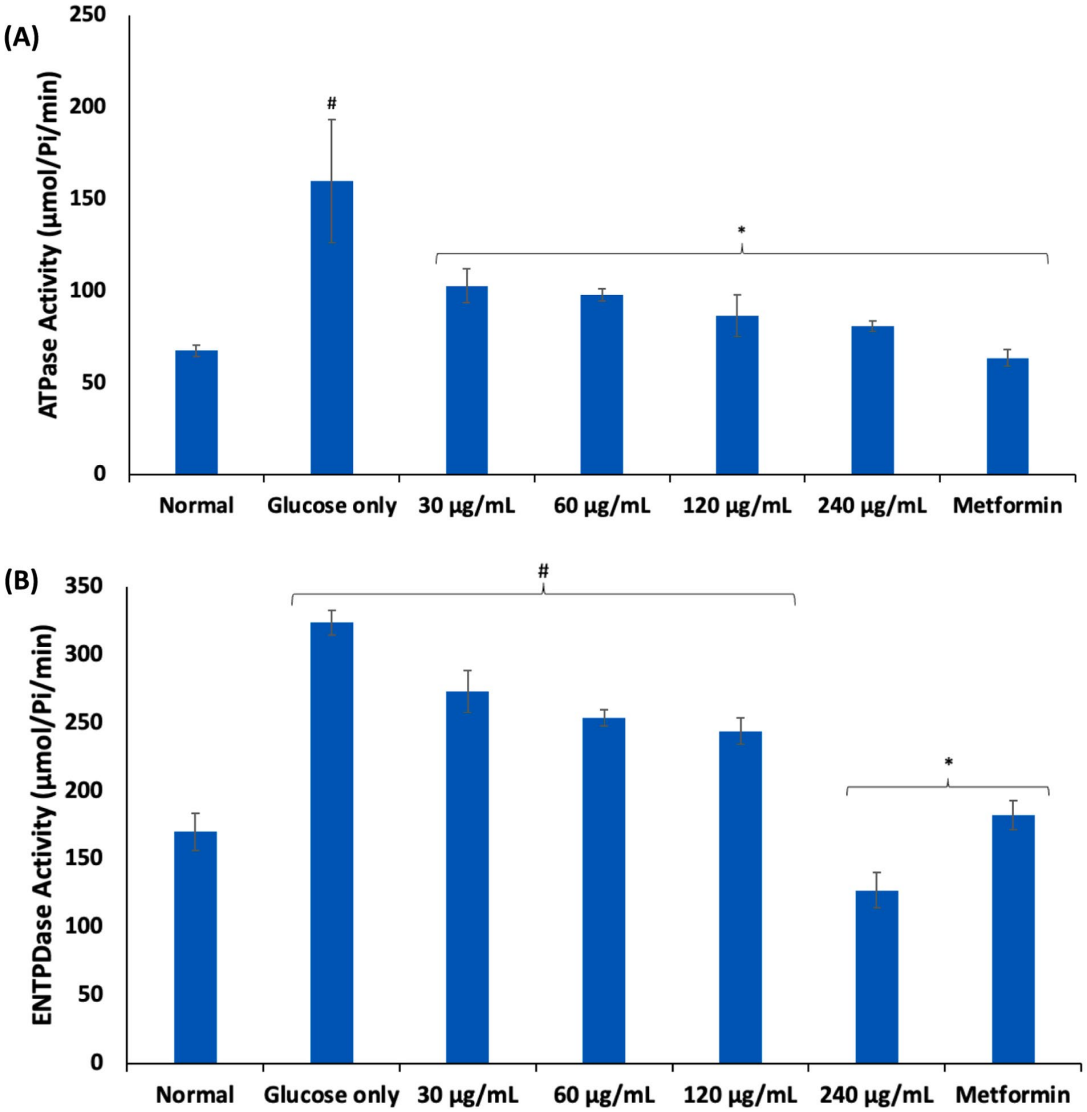
Effect of African walnut oil on **(A)** ATPase; and **(B)** ENTPDase activities in erythrocyte glucose uptake. Values = mean ± SD; *n* = 3. ^*^Statistically significant (*p* < 0.05) to glucose only. ^#^Statistically significant (*p* < 0.05) to control.

Purinergic enzyme catalyzes nucleotide metabolism leading to the hydrolysis of ATP to adenosine, which have been implicated in inflammation and immunomodulation (44). Adenosine is maintained at low levels under normal physiology. However, its high cellular levels have been reported in hypoxia and energy depletion as well as diseases such as sickle cell anemia, where they contribute to disease progression (45). As shown in Figures 5A,B, incubation of erythrocytes with glucose only, significantly (*p* < 0.05) elevated the activities of ATPase and ENTPDase. Elevation of these enzymes have been reported in glucotoxicity (15, 17, 46). These elevated activities indicate increased cellular adenosine level and reduced ATP levels, which may be a compensatory mechanism for depleted energy. Incubation with AWO, led to significant depletion in erythrocytes levels of ATPase and ENTPDase, thus, suggesting improved ATP levels and decreased adenosine levels.

### 3.5 Surface morphology

**Figure 6:**
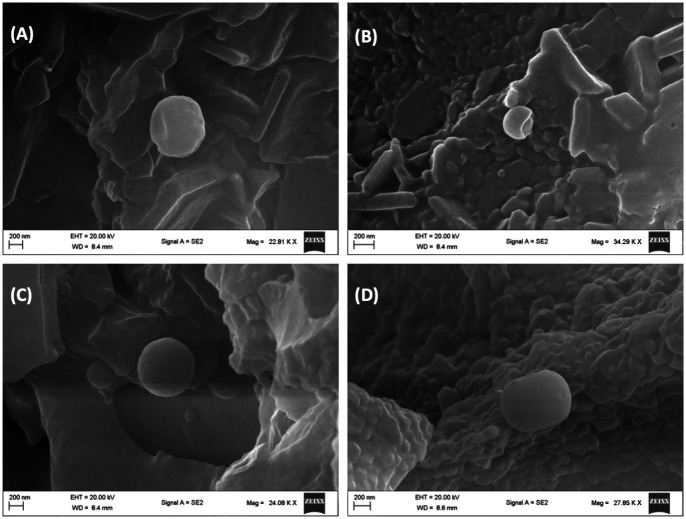
Effect of African walnut oil on tissue surface morphology in erythrocyte glucose uptake. Magnification: **A** = 22,810×; **B** = 34,290×; **C** = 24,080×; and **D** = 27,850×. **(A)** Control; **(B)** glucose only; **(C)** African walnut oil; and **(D)** metformin.

Alterations in erythrocytes’ morphology have been implicated in their dysfunction and survival. These morphological alterations have been reported in erythrocytes with impaired glucose uptake and metabolism as seen in diseases such as diabetes and sickle cell disease (1, 3, 4), where the normal physiological biconcave discoid shape is altered. As shown in Figure 6B, incubation of erythrocytes in glucose only, led to a distortion in its biconcave morphology as compared to the normal control (Figure 6A). In diabetes, this change has been attributed to hyperglycemia, oxidative stress, and reduced membrane integrity (47, 48) and has been implicated in increased blood viscosity, microvascular complications, thrombotic risks and endothelial dysfunctions (49, 50). Following incubation with AWO, the erythrocyte morphology was improved to almost near normal as shown in Figure 6C. Metformin gave the best improvement (Figure 6D). The improved morphology indicates that erythrocyte glucose uptake by AWO involves restoration of the cell’s morphology.

### 3.6 Elemental mapping

**Figure 7:**
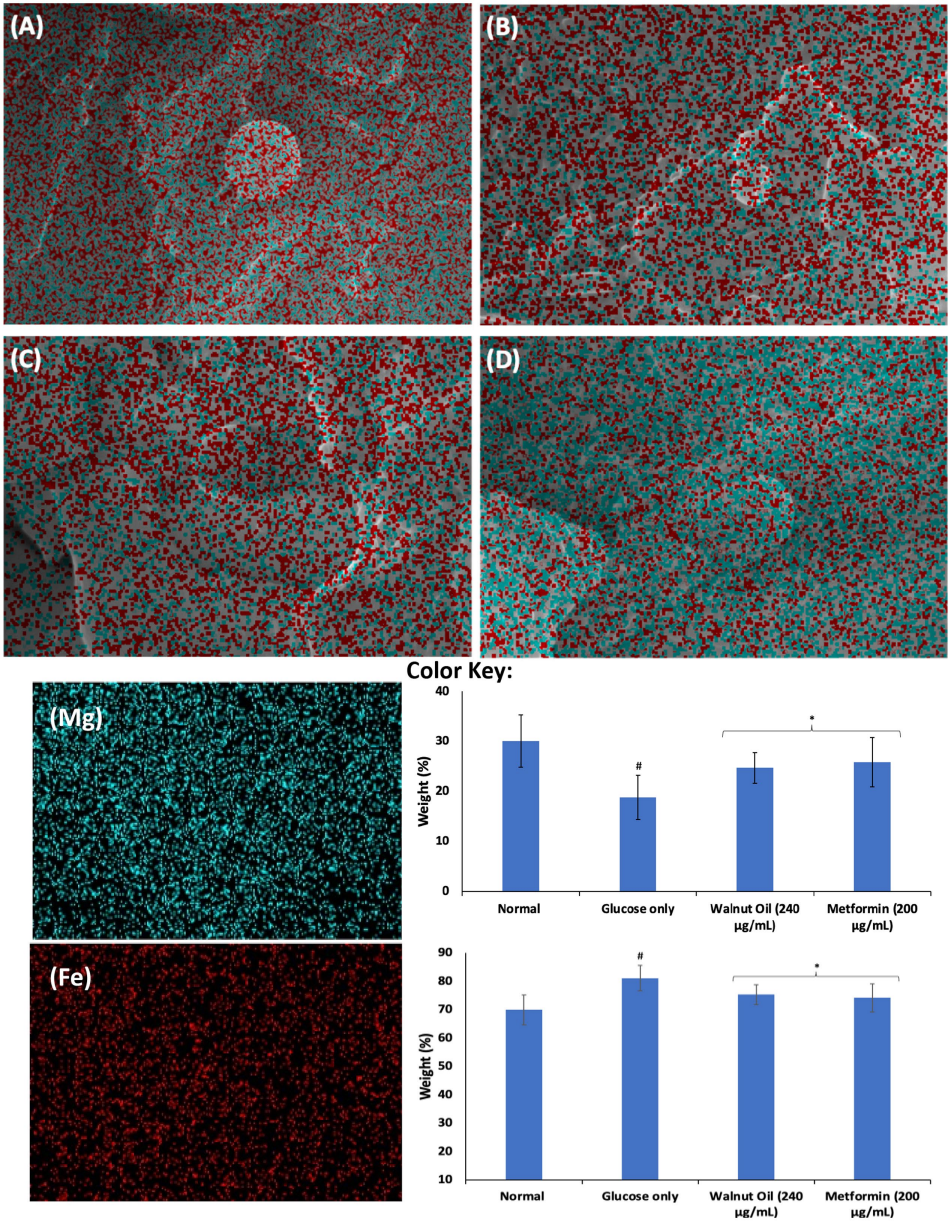
Effect of African walnut oil on tissue elemental constituents in erythrocyte glucose uptake. Values = mean ± SD; *n* = 3. Magnification: **A** = 22,810×; **B** = 34,290×; **C** = 24,080×; and **D** = 27,850×. **(A)** Control; **(B)** glucose only; **(C)** African walnut oil; **(D)** Metformin; (Mg) magnesium; and (Fe) iron.

The role of Mg and Fe in the physiology of erythrocytes have been well documented. The role of Mg in erythrocytes’ function include hemoglobin production, membrane integrity and energy production (51, 52). Its deficiency has been linked to suppressed erythrocyte energy metabolism, and has been implicated in the pathogenesis of anemia (53). Iron is an important component of hemoglobin and thus, is important in the physiology of erythrocytes (54). Its deficiency has been implicated in anemia. However, elevated Fe levels has been implicated in the pathogenesis of oxidative stress and cellular toxicity via Fenton reaction (55). As shown in Figures 7A,B, incubation with glucose only, led to significant (*p* < 0.05) depletion in erythrocyte level of magnesium and exacerbated Fe level. The depleted Mg level indicates reduced energy production and impairment of glucose metabolism, which corroborates the impaired erythrocyte glucose uptake. The elevated Fe level may indicate distortion of hemoglobin leading to release of the element, which increases the cells susceptibility to oxidative stress via Fenton reaction. Incubation with AWO significantly reversed the erythrocyte levels of Mg and Fe (Figures 7C,D). Thus, indicating an improved energy production, glucose metabolism and antioxidative activity.

### 3.7 Molecular docking

**Figure 8:**
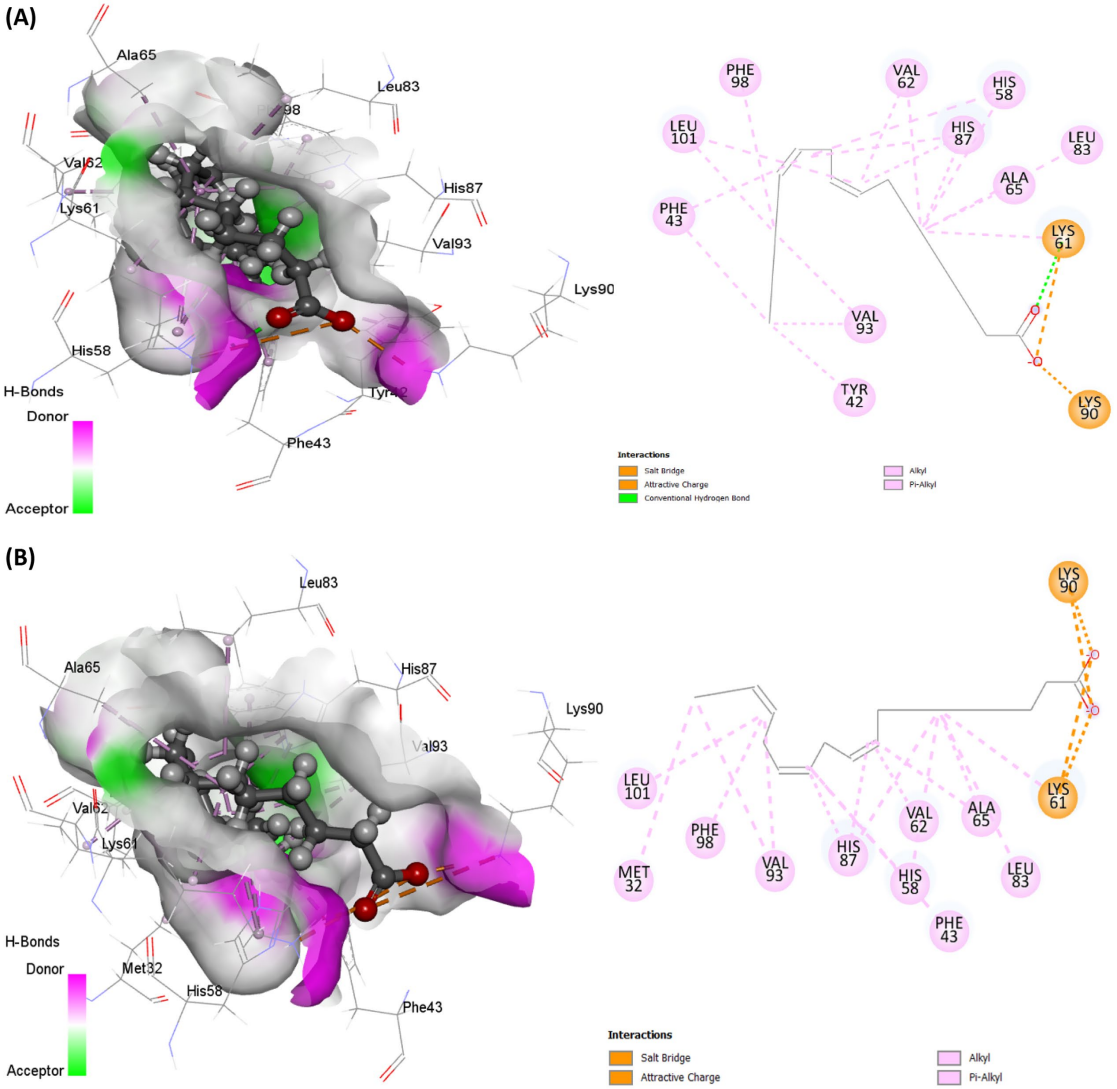
Molecular interaction of **(A)** linoleic acid and **(B)** linolenic acid with hemoglobin (PDB code: 4HHB).

Molecular docking analysis revealed strong molecular interactions of linoleic acid and linolenic acid with hemoglobin as shown in Figures 8A,B. This is further depicted by their binding energies, with linolenic acid having the lowest value (Table 1). In molecular docking, the lower the binding energy value, the stronger the interaction, thus, indicating that linolenic acid had a stronger molecular interaction and contributed more to the interaction of AWO with hemoglobin.

**Table 1:** MM/GBSA-based binding free energy profile of linolenic acid and linoleic acid bound to hemoglobin (PDB code: 4HHB).

Systems	Energy components (kcal/mol)					
	ΔG_bind_	ΔG_bindCoulomb_	ΔG_bindHbond_	ΔG_bindLipo_	ΔG_bindSolvGB_	ΔG_bindvdW_
Linolenic acid	−56.91 ± 5.28	−8.32 ± 6.70	−1.13 ± 0.59	−25.12 ± 2.88	19.79 ± 5.73	−44.42 ± 3.56
Linoleic acid	−17.68 ± 15.78	1.16 ± 9.49	−0.53 ± 0.76	−6.57 ± 6.06	2.91 ± 9.41	−15.16 ± 13.52

### 3.8 Dynamic conformational stability and fluctuations

**Figure 9:**
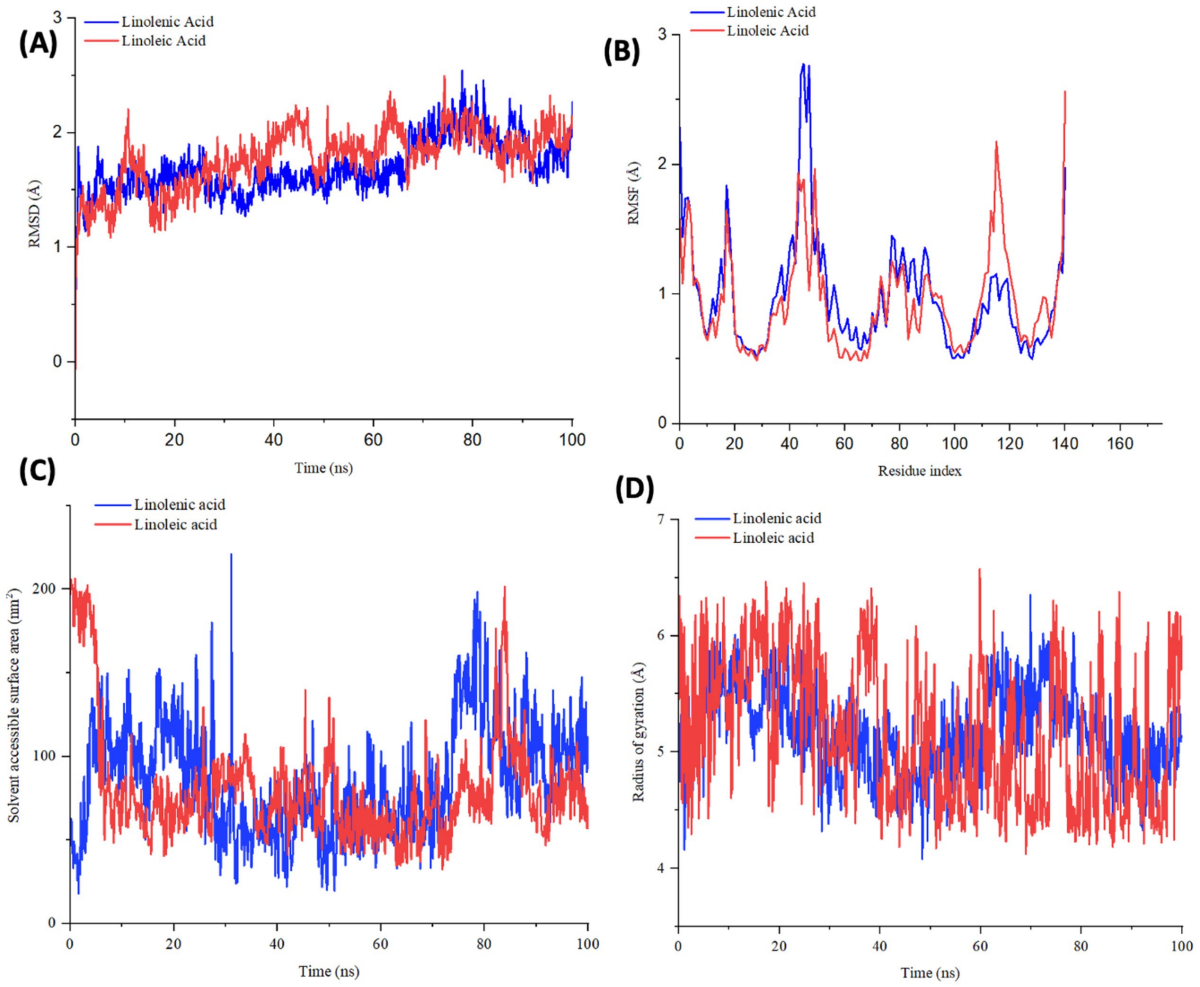
**(A)** RMSD, **(B)** RMSF, **(C)** SASA, and **(D)** rGyr profile of linolenic acid and linoleic acid bound to hemoglobin (PDB code: 4HHB).

To further demystify the observed molecular interactions, the stability and flexibility of the ligand-protein complex were subjected to root mean square deviation (RMSD) and root mean square fluctuation (RMSF) measurements via MD simulation. RMSD quantifies the disparity between a protein’s original backbone conformation from its initial position (56). The low RMSD value of linolenic acid (Table 2 and Figure 9A) indicates a potent alignment and structural stability between the omega-3 fatty acid and hemoglobin, with less deviation and conformational change. Furthermore, the low RMSF value of linolenic acid (Table 2 and Figure 9B) indicates relatively stable and less fluctuation between the omega-3 fatty acid and hemoglobin (26, 57). RMSF quantifies the deviation of atomic locations from the initiation positions with time, which defines a compound’s flexibility and dynamics (58).

**Table 2:** RMSD, RMSF, SASA, and rGyr profile of linolenic acid and linoleic acid bound to hemoglobin (PDB code: 4HHB).

Systems	Estimated average (Å)			
	RMSD	RMSF	SASA	rGyr
Linolenic acid	1.69	0.99	86.34	5.17
Linoleic acid	2.10	1.10	325.34	4.89

The compactness of linolenic with hemoglobin was further portrayed by its low Solvent Accessible Surface Area (SASA) values (Table 2 and Figure 9C). SASA quantifies how a molecule’s surface area interacts with solvents, thereby giving insights into protein folding, stability, and molecular interactions (58, 59).

However, linoleic acid had a lower radius of gyration (rGyr) value (Table 2 and Figure 9D), which indicates a rigid and compact interaction with hemoglobin. The radius of gyration quantifies the compactness and flexibility of ligand-protein complex, giving insights into the spatial conformation and protein’s diffusivity of a protein (60).

### 3.9 Protein-ligand relationship

**Figure 10:**
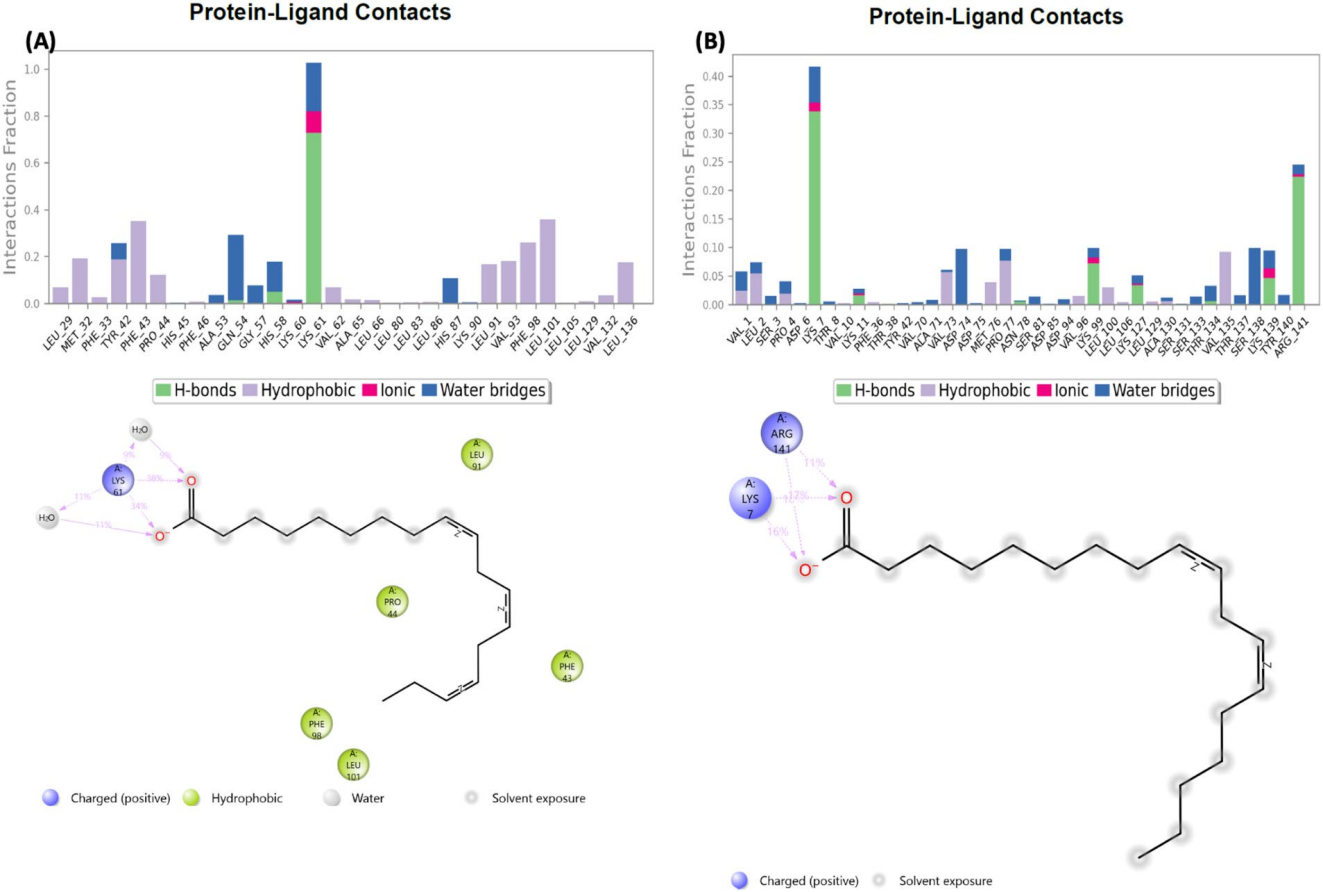
Protein-ligand contact of **(A)** linolenic acid and **(B)** linoleic acid with hemoglobin (PDB code: 4HHB).

The protein-ligand relationship between hemoglobin and AWO constituents (linolenic acid and linoleic acid) were investigated via MD simulation. As shown in Figures 10A,B, the molecular interactions of linolenic acid and linoleic acid with hemoglobin were facilitated by hydrogen (H), hydrophobic and ionic bonds as well as water bridges. The main contributors in the fatty acids to these bond interactions are their carboxyl groups (–COOH) and double bonds. Hydrogen bonding has been reported for their influence on chemical and biological reactions (61), as it allows the accurate fitting of a compound to a receptor via precise arrangement between the H-bond donor and acceptor groups (62, 63). Hydrophobic bonding between the fatty acids and hemoglobin indicates the potency and specificity of the ligand-protein complex, as the bond stimulates interactions between hydrophobic cavities within the binding sites (64, 65). The presence of ionic bonds indicates electrostatic interactions between the fatty acids and hemoglobin, and defines the former’s binding affinity and specificity (66, 67). It also portrays the orientation and conformation of the ligands within the protein binding pockets (66, 67). Water bridges are intermediaries made of water molecules which facilitate hydrogen bonding between non-interacting groups in protein binding pockets, thereby enhancing the stability of the protein-ligand complex (68–70).

## 4 Conclusion

The study provides evidence for the first time that AWO promotes glucose uptake in erythrocytes, with concomitant improvement in glucose metabolism, purinergic enzyme activities and cell morphology. AWO also demonstrated antioxidative effect by mitigating oxidative stress via suppressing SOD activity and elevating GSH level. Molecular docking and MD simulation further portrayed strong protein-ligand interactions between hemoglobin and AWO’s constituents (linolenic acid and linoleic acid). However, further pre-clinical and clinical studies are required to decipher these results and determine the translational relevance of AWO in managing diseases involving erythrocytes’ dysfunction such as diabetes.

## Data Availability

The original contributions presented in the study are included in the article/supplementary material, further inquiries can be directed to the corresponding author.

## Footnotes

^1^ www.pdb.org/pdb

^2^ https://discover.3ds.com/discovery-studio-visualizer-download

^3^ http://www.chemaxon.com
