# Correction: African walnut (*Plukenetia conophora*) oil improves glucose uptake and metabolic activities in erythrocytes

**DOI:** 10.3389/fnut.2025.1679967

**Published:** 2025-08-18

**Authors:** Ochuko L. Erukainure, Chika I. Chukwuma

**Affiliations:** ^1^Laser Research Centre, Faculty of Health Sciences, University of Johannesburg, Johannesburg, South Africa; ^2^Centre for Quality of Health and Living (CQHL), Faculty of Health and Environmental Sciences, Central University of Technology, Bloemfontein, South Africa

**Keywords:** African walnut, erythrocytes, glucose metabolism, oil, oxidative stress

In the published article, the Center for High Performance Computing (CHPC), South Africa was not acknowledged. The original statement was Authors acknowledge the assistance and support of Prof. OAT Ebuehi of the Department of Biochemistry, University of Lagos, Idi-Araba, Lagos, Nigeria, for laboratory space. The correct Acknowledgment statement appears below.

